# Medical Progress

**Published:** 1870-08

**Authors:** A. N. Bell

**Affiliations:** The 47th Anniversary of the Medical Society, of the County of Kings, Brooklyn


BUFFALO

Medical and Surgical Journal.

VOL. X. AUGUST, 1870. No. 1.

### Original Communications.

ART. I.—*Medical Progress.—An Oration, delivered by* A. N. BELL, M.D., *on the 47th Anniversary of the Medical Society, of the County of Kings, Brooklyn, April* 27, 1870.

(CONCLUDED.)

At about the beginning of the 18th century, the spirit of enquiry was severely contesting authority in everything. And in medicine particularly, numerous important facts were in process of discovery Great names had lost their potency, and inquirers after truth were gradually working into the necessity of self-reliance. Many well conducted experiments and accurately observed phenomena had been wrought out by enthusiasts in quest of shadows; but there were some reflective minds who made it their purpose to acquaint themselves with all the facts that had been adduced, for strictly utilitarian purposes. Foremost among these were Sydenham and Boerhaave. Although they were not wholly free from the hypothetical doctrines of the time, these were never allowed to gain such ascendancy over their minds as to interfere with a sound judgment. With them, experience, based on accurate observation, always took precedence of theory. Their uncommon segacity in the diagnosis of disease and discrimination of remedies, was due to a determined subordination of theories to facts, and not facts to theories.

They were both not only learned in their profession, but in its collaterals; and they possessed in a high degree the faculty of availing themselves of the knowledge of their contemporaries, and of so utilizing it as to increase the resources of medicine. With unselfish devotion and industry that never contemplated less than a knowledge of everything conducive to the successful application of their profession, they impersonated in an extraordinary degree the qualities essential to a sound progress. To Boerhaave, especially, are we indebted for the permanent revival of clinical instruction. This means of improvement was first practiced in the latter part of the sixteenth century, at the Hospital of St. Francis, Padua; and subsequently by Sylvius, at Leyden, whose clinics were held in high repute, and caused him to be regarded as the founder of this mode of instruction. But his successors let it fall into disuse for more than forty years, when it was revived by Boerhaave, who became so renowned that auditors attended him from all parts of Europe. And, owing to his success, clinical instruction was speedily established among all civilized nations.

The interval from Harvey to Boerhaave is particularly notable for the number and industry of its laborers, and the number of isolated facts brought to light. By the increase of anatomical knowledge, the publication of hospital reports, essays and treatises, operative surgery, especially, and the treatment of injuries, advanced to a high degree. Post-mortem examinations had occasionally been made from the earliest times,* but they had not earned a name. No records of such examinations seem to have been preserved until from about the beginning of the sixteenth century of the Christian Era. A. D., 1507, Antony Benivieni, of Florence, prosecuted post-mortem examinations, extending even to the practice of other physicians. Eustachius, also pursued the same course, and thus made many important contributions to anatomy. But much prejudice existed against the practice, and great perseverance was necessary to overcome it. Among those who made the most progress in this direction was Marcellus Donatus. "Let those," said he, "who interdict the opening of bodies well understand their errors. When the cause of disease is obscure, in opposing the dissection of a corpse

* Pliny.

which must soon become the food of worms, they do no good to the inanimate mass, and they cause a grave damage to the rest of mankind, for they prevent the physicians from acquiring a knowledge which may afford the means of great relief eventually to individuals attacked by a similar disease. No less blame is applicable to those delicate physicians, who from laziness or repugnance, love better to remain in the darkness of ignorance than to scrutinize laboriously, truth; not reflecting that by such conduct they render themselves culpable towards God, towards themselves, and towards society at large."*

For nearly a century subsequent, superstition outweighed the efforts of physicians to gain information by the opening of the human dead body. And although a number of medical philosophers from time to time assayed to improve their knowledge by this means there was no substantial progress. A sagacious mind was wanted who might rehearse, collate and classify the material facts that had been discovered in the progress of anatomical researches, and so generalize them as to induce truth.

Haller, the pupil of Boerhaave, trained from early age to the habit of close observation and study, entered upon the investigation of anatomical facts and physiological phenomena with a clearness of perception and judgment which led him to reject all mere matters of opinion and to receive nothing without personal verification. He pursued his investigations with characteristic patience in connection with well directed researches and experiments, and induced from the facts he had verified, irritability and sensibility as specific properties of all the muscular and nervous systems; that to either one, or both of these properties jointly, may be attributed all vital phenomena. He also traced out and discovered the process of developement of the fœtal heart and circulation, and originated the science of teratology. Iudeed, his labors contemplated a reinvestigation of all that had been previously made known in the progress of anatomy and the functions of the human organism. "It can be shown," he remarked, "even by positive calculation, that it is not possible in twenty years to work out thoroughly all parts of the human body. Animals must be dissected, but it is by no means

* Marcellus Donatus, Medica Historia, lib. IV. Renouard, p. 305.

sufficient to dissect their dead bodies; they must be vivisected. In a dead body motion is wanting. It is necessary, therefore, if we would witness motion, to observe the living animal."*

The example of Haller in carefully abstaining from all speculative opinions and of confining his researches exclusively to experiment and observation, was scarcely less beneficial than his material improvements. He, in this way, gave a new impulse to science by the spirit with which he conducted his investigations. Cullen, the cotemporary of Haller, pursued the same philosophical spirit. He estimated the properties of medicines by careful and almost skeptical observations, by which he avoided errors and inconsistencies, and distinguished the effects of remedies from physiological phenomena. His *vis medicatrix naturæ* expresses his appreciation of the specific effort of the natural powers of the organism to resist and overcome disease, and an intelligent recognition of the importance of accurate knowledge of morbid phenomena.

From Haller to Bichat was but a single step. The ancients imagined that every solid organ was reducible to what they ealled elementary fibre—a compound of earth, iron and oil, and everywhere the same. Haller's conception of this elementary fibre was, that it was to the anatomist what the line is to the mathematician. His genius endowed it with a quality only; of its substance he was ignorant. Following Haller's minute dissections, a number of investigators recognized the resemblance of certain membranes in different parts of the organism which previously had been regarded as independent structures. One of the earliest of these observers was Andrew Bonn, who published a Thesis in 1763, entitled, "*De Conditionibus Membranarum.*" Fifteen years later, Carmichael Smyth, read a paper on Inflammation, wherein he attributed the causes of the specific distinctions in the various forms of inflammation, to the differences in natural texture. He cites examples of inflammation of the mucous membranes, serous membranes, muscular fibres, etc., in each of which the inflammation is distinguishable by peculiar characters—though in different parts.† This appears to have been the earliest effort to classify disease according to the

* Preface to *Elements.*

† Medical Communications, Vol. II, p. 163

structure of the organ or tissue in which it exists. About the same time Baillie and Pinel each adopted a similar method of designating diseases according to organic structure. Insignificant as these beginnings in the study of structural anatomy now appear, they were the preludes of the most important discovery in the progress of medicine. The ideas of Bonn, Smyth, Baillie and Pinel, were seized upon by Bichat and elaborated into a substantial organic basis of a new science. Devoting himself with almost unparalled patience to the investigation of minute anatomy, Bichat sacrificed every thing else to the advancement of the object of his research. Some notion of his ardor and industry may be formed from the fact that in the short space of six months he personally examined over six hundred human dead bodies.* His indomitable energy was but the counterpart of a genius worthy of the task he undertook. Verifying all that was known before, he grasped the residue, and from it accomplished the brilliant achievement of separating the human body into its elementary tissues. And these he not only defined and described in a morphological point of view, but in detail—in their physiological functions and morbid conditions; in such wise as to render them easily recognizable by fixed properties under whatever circumstances and wherever they may be found. The elementary tissues so well described by Bichat, are the origin and foundation of Histology in all its phases, and the scientific basis of modern medicine.

For the next thirty years after the discovery of Bichat, many anatomists occupied themselves in rehearsing his researches in quest of new truths, in projecting instruments, aud in experimental inquiries and clinical observations on the functions of organic life. The examination of the elementary tissues of animals, found analagous researches into the tissues of plants. Robert Brown, Slack and others published important observations on the elementary structures and physiology of plants; and Schleiden, penetrating still further in a paper entitled "Phytogenesis,"† pointed out small sharply defined granules, generated in a granular substance surrounded by cell nuclei or cytoblasts, which he likened to granulous

---

* Notice Historique sur Bichat, Maingault, Edition of the Gen'l Anat.

† Muller's Archiv fur Anatomie.

coagulations aiound the granules. These observations were communicated to Schwann, who was struck with similar appearances in animal tissues, and thereupon conceived the idea that the same character of developement which Schleiden had discovered in plants would be found equally true of animals. From this time Histology made rapid progress, and in no other way can we so well present this most important step in medical progress, as in the words of Kolliker, one of its greatest promoters, "In the year 1838, the demonstration of Dr. Thomas Schwann of the perfectly identical cellular composition of all animal organisms, and of the origin of their higher structures from these elements, afforded the appropriate conception which united all previous observations, and a clue for further investigations. If Bichat founded Histology more theoretically by constructing a system and carrying it out logically, Schwann has by investigation afforded a basis of facts, and has thus won the second laurels in the same field. What has been done in this science since Schwann, has been indeed of great importance to physiology and medicine, and in fact of great value in a peculiarly scientific point of view, inasmuch as a great deal which Schwann only indicated or shortly adverted to as the genesis of the cell, the import of the nucleus, the developement of the higher tissues, their chemical relations, etc., has received a further development, but all this has not amounted to a step so greatly in advance as to constitute a new epoch. If without pretensions to prescience, it be permitted to speak of the future, this condition of Histology will last as long as no essential advance is made towards penetrating more deeply into organic structure, and becoming acquainted with the elements, of which that which we at present hold to be simple is composed. If it be possible that the molecules which constitute cell-membranes, muscular fibrils, axile fibre of nerves, etc., should be discovered, and the laws of their apposition and of the alterations which they undergo in the course of their origin, the growth and the activity of the at present so-called elementary parts should be made out, then a new era will commence for Histology, and the discoverer of the law of cell-genesis, or of a molecular theory, will be as much or more celebrated than the originator of the doctrine of the composition of all animal tissues out of cells. * * * * *

As regards the general positions of Histology, the science has made no important progress since Schwann; however much has been attained by the confirmation of the broad outlines of his doctrines. The position that all the higher animals at one time consist of cells, and develope from them higher elementary parts, stands firm, though it must not be understood as if cells, or their derivities were the sole possible or existing elements of animals. In the same way Schwann's conception of the genesis of cells though considerably modified and extended, has not been essentially changed; since the cell-nucleus still remains as the principal factor of cell-development and of cell multiplication. Least advance has been made in the laws which regulate the origin of cells and of the higher elements; and our own acquaintance with the elementary processes which take place during the formation of organs, cannot be regarded as very slight. Yet the right track in clearing up these points has been entered upon; and a logical investigation of the chemical relations of the elementary parts, and of the molecular forces after the manner of Donders, Dubois, Ludwig, and others, combined with a more profouud microscopic examination of them, such as has already taken place with regard to the muscles and nerves, and further, a histological treatment of embryology, such as has been attempted by Reichert, Vogt and myself, will assuredly raise the veil and bring us step by step nearer to the desired, though, perhaps, never to be reached end."*

Of the *nature* of disease and its discovery, the ancients for the want of anatomical knowledge regarded all morbid phenomena or symptoms as evidences of something that had entered into or grafted itself upon the body. Hence their treatment consisted in an effort to dislodge it, and chiefly consisted in the use of evacuants. For this reason the introduction of Peruvian bark and some other useful remedies in the treatment of disease were opposed on the ground that they produced no sensible evacuation, and were therefore inconsistent with accepted theory—that no disease was curable without the expulsion of bile, phlegm or other humors. The Stahlites regarded fever as a natural and salutary effort of the soul to free itself from an injurious substance; to arrest it was contrary

* Introduction to Manual of Human Histology.

to the vital principle and therefore likely to do more harm than good. The Arabians held that small pox was innate to man; and therefore to prevent its development was to oppose the action of nature, and to keep the enemy in his place. By the improvement of modern times, disease is known to consist of an intrinsical change in the structure of the organism. The name of a disease may, and usually does express some important fact or characteristic, but this is only an integral part of the existing change. The symptoms present indicate the nature of the changes produced and these changes (not the symptoms), constitute the disease. And, inasmuch as no single organ can undergo a change of structure, or function depart from its healthy standard, without corresponding changes in all the rest, it is apparent that the foundation of the medical art must be laid in an accurate knowledge of the structure and functions of the human body. It may be safely stated in this connection, that not a single remedy for any disease whatever, has ever been discovered by following a theory or hypothesis. But, on the contrary, as truly remarked by Virchow, "the history of medicine teaches us, if we will only take a somewhat comprehensive survey of it, that at all times permanent advances have been marked by anatomical innovations, and that every more important epoch has been directly ushered in by a series of important discoveries concerning the structure of the body.*" The maxim that "knowledge of disease is half its cure," was appreciated, however, though it may not have been expressed, even before the era of Histology. Pathology had already involved the fundamental art of diagnosis as the foundation of all enlightened practice. But the means of exercising the art of diagnosis were far more limited; and the practical results correspondingly inefficient.

The eighteenth century closed with a concurrence of effort on the part of a number of individuals all tending to the same goal—to lift the medical art out of the conjectural hypotheses of ages and establish it upon a scientific basis. The great achievement of Bichat, as already shown, was not accomplished single-handed. Others had begun to clear the way. The young giant quickly ran his brilliant career, but left behind him worthy followers. Laennec, the pupil

* Cellular Patology.

of Corvisart, while attending his master's lectures at La Charitè, became strongly impressed with the importance of discovering internal lesions by external signs. As early as 1763, Avenbrugger, a German physician, had introduced percussion as a means of diagnosis, but it had been rejected by the profession and was not again revived until by Corvisart, thirty years afterwards. Boyle, a fellow student with Laennec, applied his ear to the chest, while attending Corvisart's lectures. Laennec followed suit, and conceived the idea of increasing his powers of discrimination by artificial means. May 14, 1815, fifteen days after having read a paper on his favorite study (before the Societe de l'Ecole), he added the stethescope to his means of diagnosis. He was shortly afterwards appointed to the Hospital Beaujon, and soon after to the Necker, which afforded him abundant means for the cultivation of his ear, and the verification of his diagnosis. After three years assiduous study, he published his treatise on Mediate Auscultation. Louis, Andral, Cruveilheir, Meckel, Abercrombie, Mayo, Hope, Carswell, and others progressed in the same direction as Laennic, by diligently studying all the functions of the human body during life, and examining all the organs after death.

Bichat condensed anatomical knowledge into a grand reservoir that ever since his day has been overflowing its banks and fertilizing a continuously expanding field of scientific culture.

Willis, Cabanis, Camper, and other collaborators of Bonn, Smythe, Pinel and Bailie, investigated the nervous system, and were the first to regard the brain as the viscera of the understanding, with special functions in communication with different parts of the organism. The first to discover a difference in the functions of the different roots of the spinal nerves, was Alexander Walker;* and shortly after him, Sir Charles Bell published his "Idea of a New Anatomy of the Brain," showing a difference between the nervous elements employed in the different functions of the nervous system. Subsequently he showed that the nerves of motion were distinct from those of sensation, and *suggested* that the posterior or ganglionic roots of the spinal nerves are nerves of sensation, while the

---

* Archives of Universal Science, 1809.

anterior roots are nerves of motion. This suggestion was taken up and first claimed by Magendie, and subsequently adopted by a number of anatomists, who have since demonstrated that the difference in the nerves of motion and nerves of sensation as first discovered by Bell, finds its true distinction in the grey and white matter;—the former being the principal conductor of the sensitive impressions, and the latter, impressions of motion. The demonstration of this truth, and still further the decussation of the conductors of sensitive impressions in the spinal cord, and the decussation of the conductors of motive impressions in the medulla oblongata, with elaborate pathological conclusions, have been the result of vivisections and clinical observation.

The discovery of physiological tissue genesis by Schwann, was followed by the still more profound researches of Johannes Muller* establishing pathology on the same basis; in determining the fundamental law of similarity between pathological and physiological tissue development, which has since been so abundantly verified by Wedl, Virchow and others. Vogel, Lebert, Rokitansky, Paget and others, have elaborated morbid Histology in the same vein. Meanwhile the *free* cell-development of Schwann or blastema formation, has been investigated anew by Reichert Henle, Maudl and Remak; the last of whom in 1852, declared *free* cell-development an error, and announced "*Omnis cellula in cellula,*"* as the true conception of cell growth. Two years later Virchow echoed the same doctrine in "Omnis cellula e cellula,"† which is now well nigh the accepted basis of cellular pathology. Virchow's writings are so recent and of such easy access that an attempt to state his doctrines is unnecessary. With a profound respect for the past, he uses the deficiencies of his predecessors as means of improvement for himself, while he enhances their excellences by presenting them in a more favorable aspect. Virchow's labors from the first have been characterized by the same generous spirit. A truth was no sooner known to himself than it was communicated to the profession. And his name is identified in the progress of medicine for the last twenty years to a degree scarcely equalled by any other. By this means his discover-

* Bau der Krankhaften Geschwulste.
* Muller's Archives, 1852.
† Beitr. 2 Spec. Pathologie und Thuapie, 1854.

ies and views have been subjected to examination from all quarters. The result has been that when the time came for him to consolidate his writings, as in the several volumes which he has published during the last ten years, he had the benefit of all his collaborators, and he has so compacted their and his own labors together, and so connected them with the past as to present the sum of anatomical improvement acquired during the present century.

The sum of medical progress now rests upon :—

(1.) An *anatomy,* which in a descriptive point of view is perfect and thoroughly worked out, and structurally nearly so.

(2.) A *Physiology* comprehending not only an accurate knowledge of the functions of the chief organs and tissues which constitute them, but of the molecules of less than one twenty-thousandth part of an inch in diameter, and almost the process of their development. The growth, contractility and movements of the living molecules, being demonstrable by means of the microscope with the same degree of accuracy as the largest cells and fibres; and these molecules are known to possess independent vital actions,—to produce nuclei, cells, fibres, tubes and membranes which unite and form the various tissues and organs of the body.

(3.) A *Pathology,* which determines after death the relations of morbid conditions and the symptoms of the diseases that cause them, in the same manner as the healthy body is explored with a view to a knowledge of its structure—and no less completely. Indeed as descriptive anatomy is perfect, to the same degree is pathology—uniting with anatomy and physiology, to constitute *Histology,* the highest mark of medical progress.

(4.) A *Diagnosis ;*—Aided by the Microscope, Stethescope, Laryngoscope, Opthalmoscope, Endoscope, Spectroscope, Thermometer, Sphigmograph, Dynamograph, and various other speculi and instruments, by means of which the chemico-physiological and pathological changes are studied and recognized. Elementary forms and deposits, amorphous granules, crystaline structures, simple and organized cells—capable of growth or otherwise; granules fibres and compound corpuscles; exudations of every degree of consistence; pigments of various shades—all, in their healthy or morbid states, discoverable, countable, definable. No amount of professed experience merely, exclusive doctrine or speculative theory can withstand the logical facts elucidated by these means. And they not only doom to inevitable oblivion existing fallacies, but they are a bulwark for the future. Standard specimens of organic forms which compose all the textures of the human body, are now within the reach of every student. And most of the recent works on practical medicine, surgery and histology, are illustrated by the arts of the engraver with what may be deemed standards of comparison for verifying the accuracy of observations by the aid of instruments.

Chemistry and Philosophy, are also instruments of diagnosis. By means of these, plants and animals are transformed in all stages of growth and development; the relations between them and the atmosphere determined; the nature of all substances—organic and inorganic, solid, liquid, or gaseous—are ascertained with precision. Physical and vital actions, chemical, electrical and mechanical influences—are utilized in diagnosis, and among the means at our disposal.

THE PRESENT STATUS OF MEDICINE, and the means by which it has been attained, distinctly point to the source of all future progress. It is in the field of exact scientific investigation into questions and problems which the most recent advances have opened to view. Each new fact, patiently grounded on exact knowledge and established beyond question, though it may for a time appear to be an idle and useless addition, may eventually fructify into some useful generalization applicable in the most unexpected and startling manner to the prevention or cure of disease, and the promotion of human happiness.

### THERAPEUTICS.

THE ART OF THERAPEUTICS, comprehending the treatment of disease is commonly considered the rear rank of medical progress. With a brief glance at this, and I will tax your patience no longer. It should be borne in mind that this branch of medicine is not only the most difficult, but that it is, rationally, at least, always the junior. When we reflect upon the number of asserted remedies, the pretended discoveries, the healing arts, the certified effects and the grave-yard certificates of universal cures as the fruit of credulity and other obstacles with which we have to contend; and when we consider how recently the auxilliary sciences of Botany and Chemistry have made their chief advances; the still imperfect state of meteorology, and other sciences and arts upon which our knowledge of therapeutic means so much depends—the wonder is that the uncertainty is not much greater than it is, instead of being marked, as the relative progress of therapeutics certainly is, by the most signal triumphs in the history of medicine. The art of therapeutics consists in the application of natural and artificial products from all sources to the preservation of health and the cure of disease, hence this branch of medicine must of necessity always remain incomplete.

Many remedies known to be directly curative of certain diseases, such as cinchona and its salts, sulphur, mercury, cod-liver oil, lemon juice, etc., are the result of empirical observation. And so too with regard to the specific effects of such agents as anæsthetics and vaccinia. By accident or experiment, it is discovered that a certain substance is of use in a particular disorder. The same remedy is subsequently and repeatedly administered in like condition, and upon a number of such data an empirical system is established. This kind of practice is in accord with the vulgar acceptation of the practice of medicine, and requires but little knowledge. Those who accept it as the sum of medical knowledge stand in the same relation to modern medicine as the ancients. And by a precipitation of judgment common to the unenlightened, they assert remeedies of whose properties they know nothing—for diseases of which they are equally ignorant, and call themselves physicians. But however limited the knowledge, and extensive the danger of such practitioners, the physio-pathological phenomena in the application of remedies, empiracally, are valuable, because their utility is made known by therapeutical proof. Indeed every effort to alleviate human suffering, however humble, is worthy of the attention of the enlightened physician. And we should remember that the powers of mankind in this direction are not wholly limited to the votaries of science. Improvements have frequently been derived from most humble sources, or seized upon under the most fortuitous circumstances. And medical progress as a whole, is the result of the succesful labors of many individuals. While a few persons may be identified with certain improvements of their time, it is none the less true, as a general rule, that such persons deserve credit for only a part of the progress made under their names. Indeed, silent workers often render the most efficient service, confirming or refuting the published accounts of the few. The final establishment of important truths are usually to be recognized in the co-ordinate experiences of many observers. And every single step in the direction of prevention and cure, is progress.

Referring to modern improvements, in an address before the British Medical Association, recently, Sir William Jenner makes the following remarks:

"Who that has suffered from a painful local affection can think of the alleviation of his sufferings which follows from the subcutaneous injections of an anodyne, without gratitude? Who is there that has had to submit to the knife of the surgeon whose heart does not overflow with gratitude to those who introduced and perfected anæsthesia? The electric telegraph, the greatest marvel of our time, was a thing which in a rough way scientific men had long thought possible; but to cut for stone, and to know nothing of the agony, to have a leg removed and smilingly to ask when the operation is over—when are you going to begin?—these are marvels of which no one dreamt; no exaggerations of fiction equal this reality. The discovery of the value of subcutaneous injection of anodynes, and local anæsthesia by ice, ether spray, and of general anæsthesia by ether, chloroform, and nitrous oxide, are advances in alleviate medicine worthy to rank with the advances in preventive, curative and prolongative medicines."

It well becomes us to be cautious in the adoption of new remedies, and to accept those only which will admit of logical conclusions, based upon accurate knowledge of the nature of the remedy and the state of the organism at the time; involving an accurate knowledge of both structure and function. With such knowledge there is quite as much certainty in medicine as in any other science or profession. And he who reproaches physicians with differing more than other men in identical pursuits, or charges them with being more uncertain in their conclusions than other professions—he who asserts or accepts such propositions has neglected his education. Physicians are far from assuming that their profession is perfect. Well do they know indeed, that the pathway to scientific truth is beset with many doubts and clogged with incessant obstacles. The enjoyment of health and long life are recognized by all men as among the greatest boons of human aspirations. The best way to promote these blessings—how to relieve pain, to cure and to prevent disease—are objects worthy of the highest ambition and of the noblest contest. And we maintain that in the exercise of these efforts as a profession, no human pursuit surpasses medicine, either in the certainty of its conclusions or in its positive and increasing blessings to mankind. What, indeed, are the certainties and emblems of progress in other professions and sciences that the reproach of being uncertain and stationary should specially apply to medicine? The law, we are told, is the perfection of human reasoning—its doctrines are confined to questions of right and wrong; in which the whole moral faculties of man instinctively lead the judge to decide aright. Besides, lawyers and judges render their opinions with the utmost deliberation after the amplest opportunity for research. But where are the clients who are satisfied with the unerring opinions of lawyers or the unappealable judgments of courts? The law pleads defectiveness of evidence in extenuation of such uncertainties. But is there any one within my hearing that does not know that the interpretations of organic law, which are wholly free from such questions, are not equally uncertain? Physicians are expected to be in a constant state of readiness to decide upon the most difficult question's at a moment's warning—but who that is informed on these matters will say that their decisions are less certain than the lawyers? Of statesmen;—on manufacturing, tariffs, internal improvements, educational systems, citizenship, banks, legal tenders and gold payments—are opinions unanimous and certain on these questions? Mathematics—that certain science from which engineers and architects with the amplest data for the most precise investigation, are they all of one opinion on the great East River Bridge, or other like structures? And, how will physicians compare with theologians? Are these latter all agreed on the most direct road to the Celestial World? or even on the meaning of words and the interpretation of phrases derived from the same sources of knowledge; or on confessions of faith, administration of ordinances and church government? And, as to being stationary.—Early in the history of mankind, physicians were of those only who occupied the highest social status, and did most for the promotion of the general welfare. And the middle ages were lighted up by stars devoted to medicine, whose constellations uncovered the darkness of superstition. Physicians in all ages have attended at the birth of philosophy and learning—have nurtured them in youth, maintained them in manhood and supported them in declining years. In the progress of modern civilization which took its rise some four centuries ago, who among the learned men of the time excelled Linacre?

We have already shown that Bacon had his peer in Harvey. Newton in the discovery of the universal law of gravitation, also had his cotemporary and equal in Haller, who discovered the laws and special forces of organic life. An hundred years ago the law gave to enlightened nations a Blackstone, but medicine gave them a Jenner! And what language is there that can supply the words to express the blessings he conferred on mankind! The beginning of the present century produced many statesmen, philosophers and scientists—but who among them all is comparable to Bichat! And more recently, of the discovery and application of anesthetics—peerless and alone! The whole world, I fear, will long remain our debtor. Medicine uncertain and stationary indeed! These are no gleanings. But only a few of the stars of the first magnitude in the midst of a galaxy that illumine the civilization of mankind the world over.

